# Influence of Atomic Doping on Thermal Stability of Ferrite Nanoparticles—Structural and Magnetic Studies

**DOI:** 10.3390/ma14010100

**Published:** 2020-12-29

**Authors:** Urszula Klekotka, Dariusz Satuła, Simo Spassov, Beata Kalska-Szostko

**Affiliations:** 1 Faculty of Chemistry, University of Białystok, Ciołkowskiego 1K 1, 15-245 Białystok, Poland; u.klekotka@uwb.edu.pl; 2 Faculty of Physics, University of Białystok, Ciołkowskiego 1L, 15-245 Białystok, Poland; d.satula@uwb.edu.pl; 3 Laboratory for Environmental Magnetism, Geophysical Centre of the Royal Meteorological Institute of Belgium, 5670 Dourbes (Viroinval), Belgium; simo@meteo.be

**Keywords:** ferrite, nanoparticles, high-temperature corrosion, oxidation

## Abstract

In this paper, a series of experiments are reported where ferrite nanoparticles were synthesized with different substitution percentages (5, 10, 15, or 20%) of Fe^2+^ by Co^2+^, Mn^2+^, or Ni^2+^ ions. Afterwards, the prepared nanoparticles were thermally treated between 50 and 500 °C in air for 24 h in order to observe how doping influences the oxidation process induced by temperature elevation and access to O_2_. Nanoparticles were imaged before and after thermal treatment by transmission electron microscopy and were analyzed by X-ray diffraction, vibrating sample magnetometry, and Mössbauer spectroscopy. Presented studies reveal that the amount and kind of doped transition metals (of replaced Fe^2+^) strongly affect the oxidation process of ferrite nanoparticles, which can govern the application possibility. Each transition element suppresses the oxidation process in comparison to pure Fe-oxides, with the highest impact seen with Ni^2+^.

## 1. Introduction

Forecasted multifunctional applications cause great interest in the development and testing of different magnetic nanomaterials. Due to variable properties, they can be used in information technology such as memory storage media [1], magnetic recording [2], sensors [3], and logical devices [4]. Another important target for scientists is in the field of medicine, where nanomaterials can play various roles from drug delivery media [5,6] to sensing materials in biological composites and sensors [7,8] or positive and negative contrast agents in magnetic resonance imaging (MRI) [9]. Huge attention has also been focused on living cell treatment via drug delivery [10] or magnetic hyperthermia [11] with the use of functional magnetic particles. Nanometer-sized magnetite-based particles, for which a superparamagnetic effect is expected, are desirable as an efficient thermal center and further anti-cancer therapy media. The most required structures are those which show multifunctional aiming activity. Coexistence of sufficient magnetic response in an alternating magnetic field with locally toxic leakage of non-inert elements, such as Co, can enhance the strength of medical treatment. The ferrofluids obtained from modified (i.e., doped with *3d* metals) magnetite (Fe_3_O_4_) [12], maghemite (γ-Fe_2_O_3_) [13], or hematite (α-Fe_2_O_3_) [14] exhibit changes in the ferromagnetic (*senso lato)* response of nanoparticles. Thermal effects induced by alternating (AF) magnetic fields and modulated by field frequency are dependent on the type, shape, and size of the particles [15]. Therefore, various nanoparticles exposed to AF fields will release a different amount of heat due to re-magnetization processes, based on the Néel effect (changing of magnetic moment direction without particle movement [16]). Its value depends on the aforementioned properties. Any structural or morphological modification of toxic systems results in a curing effect or general cytotoxicity [17].

Before nanoparticles can be applied to magnetic hyperthermia, they have to be properly prepared. Synthetic fabrication of fine nanomaterials (<50 nm) with the desired properties is not an easy task, since homogenous and stable samples are not so simply obtained. Chemical procedures need to deal with the problems of ideal pureness (single phase) of the obtained structures. Only very restricted synthetic methods can eliminate possible impurities, which include oxidation products that modify a nanoparticle system and its magnetic properties greatly [18]. Therefore, the effect of Fe^2+^ substitution on chemical/thermal stability, minimizing the oxidation process, is worth studying. On the other hand, application of nanostructures as magnetic recording media also requires high thermal stability to preserve the magnetic state for a long period of time.

It was observed that one of the important factors of nanoparticles’ interaction with external magnetic field is hematite formation at the surface of magnetic nanoparticles (MNPs). Even the presence of the smallest amounts of α-Fe_2_O_3_ on their surface causes an extraordinary orientation of magnetic moments in the presence of an external magnetic field, which results in the superparamagnetic fluctuation being suppressed [19]. It was reported that the surface anisotropy of nanoparticles modified by hematite causes a nonparallel arrangement of magnetic moments on the surface of nanoparticles. The presence of such layers influences the MNP response in an external AF field and is directly related to the heating capabilities of the particles. Substitution of magnetite or other iron oxides with elements such as Zn, Gd, Eu, and Y not only influences the magnetic properties of the nanoparticles but also reflects on the specific absorption rate (SAR) and intrinsic loss of power (IL), for example [20,21,22]. Zn-doped magnetite nanoparticles exhibit high magnetization values and, therefore, are used in high-density magnetic storage, radar absorbents, photocatalysis, and drug delivery systems [23]. Yttrium and other lanthanide ions have unique optical features and increase magnetization of. for example. magnetite nanoparticles [22].

Gentle heat treatment in specific conditions can also lead to the formation of very unique Fe-oxide forms, such as ε-Fe_2_O_3_ or its derivatives. The presence of this structure influences magnetic properties of the system [24]. Ferrite nanoparticles with the general formula MFe_2_O_4_ (where M = Co, Mn, Ni) are widely described in the literature and their potential applications have been discussed [25]. They can be obtained by both hydrolytic (co-precipitation, hydrothermal co-precipitation, and reversed micelles co-precipitation) and non-hydrolytic methods [26]. Every technique has its advantages and disadvantages; therefore, all these methods need to be implemented in special conditions. In this paper, particles with the composition M_x_Fe_3−x_O_4_ were prepared by co-precipitation of Fe^2+^ and Fe^3+^ chlorides in a basic environment. This synthetic technique allows obtaining ferrite nanoparticles dispersible in water, easy to be functionalized, with size range of 5–50 nm. The purpose of preparing such nanoparticles was to describe their thermal resistivity against an oxidation process induced by heating in a wide temperature range (50–500 °C). Magnetic properties characterization of thermally treated nanoparticles was carried out along with estimation of their phase change. 

## 2. Experimental

### 2.1. Materials and Apparatus

In order to obtain Co-, Mn-, and Ni-doped ferrite nanoparticles, the following chemicals were used: iron (III) and (II) chlorides, cobalt (II) chloride, manganese (II) chloride, nickel (II) chloride, ammonia solution (POCH), and tetrabutylammonium hydroxide (TBAOH) (Sigma Aldrich, Darmstadt, Germany). Cleaning and separation of nanoparticles was performed with acetone, a sonication bath, and a permanent magnet.

The synthesized ferrite nanoparticles were characterized by X-ray diffraction (XRD) using the Agilent Technologies SuperNova diffractometer (Yarnton, Oxfordshire OX5 1QU, UK) with a Mo micro-focused source (K_α2_ = 0.713067 Å); transmission electron microscopy (TEM)—FEI Tecnai G2 X-TWIN 200kV microscope (Thermo Fisher Scientific, Hilsboro, OR, USA); infrared spectroscopy (IR) (Nicolet 6700 spectrometer working in reflecting mode, (Thermo Fisher Scientific, Hilsboro, OR, USA); vibrating sample magnetometry (SQUID MPMS3, QuantumDesign, California, CA, USA); Mettler Toledo Differential Scanning Calorimeter (DSC, Mettler Toledo, Columbus, OH, USA) with the STAR system. Mössbauer spectroscopy (MS) was performed using a spectrometer working in a constant acceleration mode with a CoRh radioactive source. Metallic iron foil (α-Fe) was used as reference material. All samples were measured in the transmission mode. Elemental composition was inspected using an energy dispersive X-ray spectrometer integrated into a scanning electron microscope (INSPEC 60, (Thermo Fisher Scientific, Hilsboro, OR, USA)).

### 2.2. Synthesis of Ferrite Nanoparticles

In the present paper, ferrite nanoparticles containing four concentrations (5%, 10%, 15%, and 20%) of Co^2+^, Mn^2+^, or Ni^2+^ elements were prepared. A typical Massart-like method [27] was used for the MNP synthesization. This routine is based on co-precipitation of Fe^3+^ and Fe^2+^ chlorides in ammonia aqueous solution at a temperature of 80 °C under an Ar atmosphere, where TBAOH was used as a surfactant. To obtain ferrites with the desired concentration of Co^2+^, Mn^2+^, or Ni^2+^, the Fe^2+^ ions were replaced partly (5%, 10%, 15%, or 20%) by a proper M^2+^ salt. Finally, the nanoparticles were separated from the solution with a handheld permanent magnet, washed in deoxygenated acetone, and dried to powder form in a vacuum evaporator. Table 1 provides an overview of the obtained nanoparticles and their abbreviations used throughout the paper.

The obtained nanoparticle powders were thermally treated in an oven for 24 h in air and at selected temperatures (temperature range 50–500 °C, with 50 °C steps, or only at 500 °C) [28]. After thermal treatment, the powders were characterized by physicochemical methods in order to analyze the effect of oxidation. 

The elemental analysis confirmed the requested composition of the nanoparticles and proved the adequacy of the employed protocol for the fabrication process of nanoparticles on demand.

## 3. Results and Discussion

### 3.1. Transmission Electron Microscopy (TEM)

**Figure 1:**
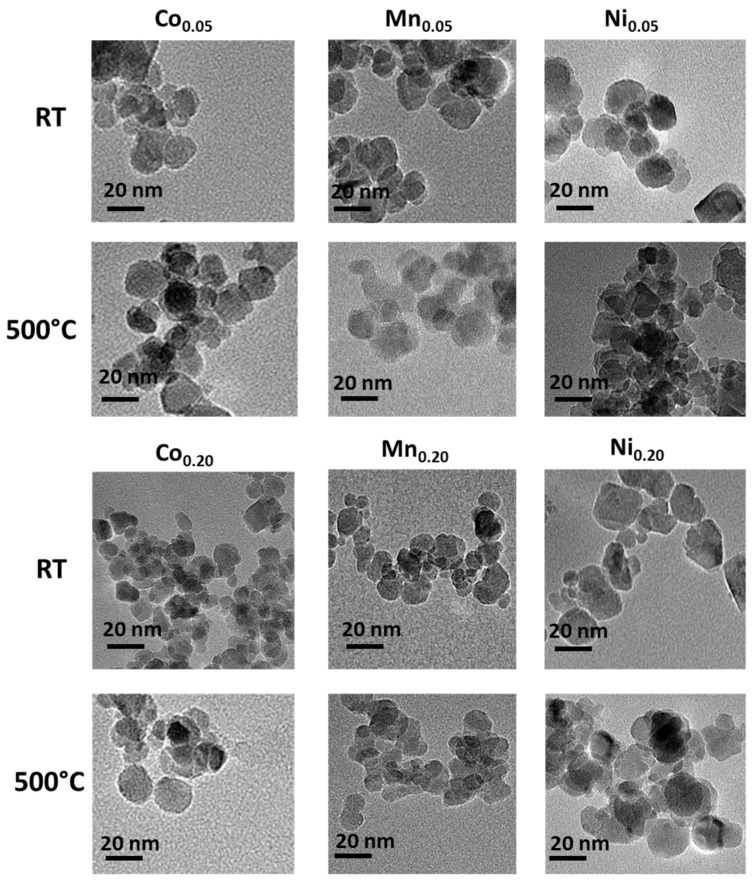
TEM images of as-prepared (RT) and thermally treated (at 500 °C) ferrite nanoparticles. Only the series of 5% and 20% of Co, Mn, and Ni are presented. The exact composition is given in Table 1.

Qualitative analysis of the MNP morphology before (RT–room temperature) and after temperature treatment (500 °C) was performed by TEM. For this purpose, selected images of each type of particles were taken. This method allows for the determination of shape, diameter, and size distribution of the observed objects. To present trends in the series, only selected images were chosen to be depicted in Figure 1.

The images depicted in Figure 1 show that the particles are rather round in shape regardless of the doping element and its concentration. The size distribution is not very broad before and after the heat treatment as well. Neither increased tendency for aggregation nor significant particle growth is observed as possible consequence of temperature rise [28].

### 3.2. X-ray Diffraction (XRD)

X-ray diffraction on a small amount of nanoparticles placed on a nylon loop was performed on all particle samples before and after 24 h heating at every defined temperature.

**Figure 2:**
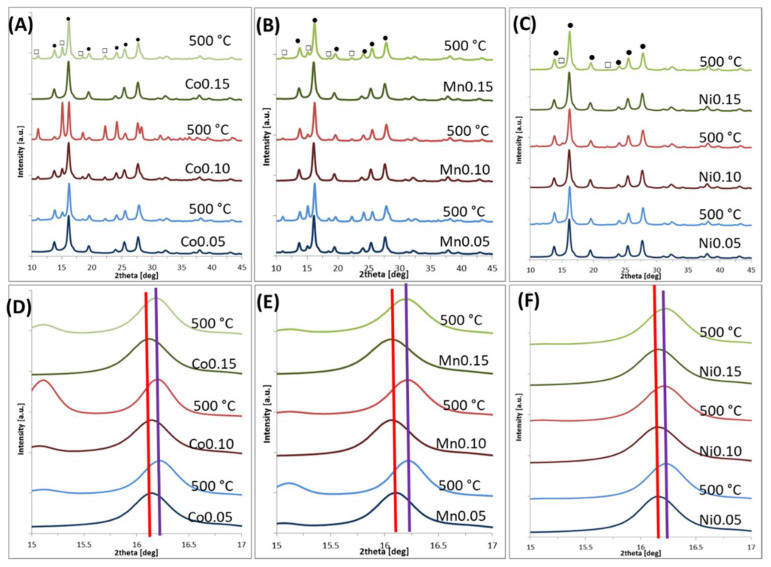
X-ray diffraction patterns of nanoparticles before and after temperature treatment (at 500 °C): (**A**) Co_0.05_, Co_0.10_, Co_0.15_; (**B**) Mn_0.05_, Mn_0.10_, Mn_0.15_; (**C**) Ni_0.05_, Ni_0.10_, Ni_0.15_. Note: ● Fe_3_O_4_ signals, □ α-Fe_2_O_3_ signals. (**D**–**F**) Respective series zoom on peak (311) of magnetite structure.

**Figure 3:**
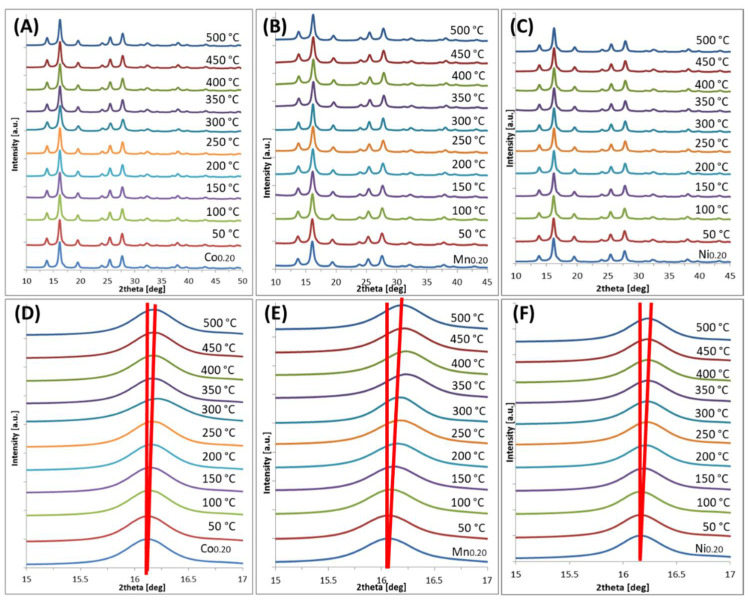
X-ray diffraction patterns of nanoparticles before and after temperature treatment (50–500 °C): (**A**) Co_0.2_; (**B**) Mn_0.2_; (**C**) Ni_0.2_; (**D**–**F**) Respective series zoom on peak (311) of magnetite structure.

**Figure 4:**
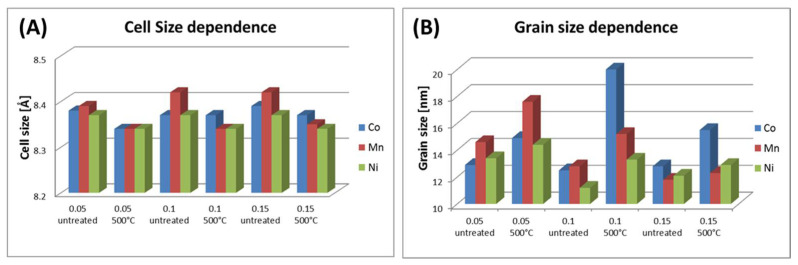
Bars plot of nanoparticles’ (**A**) cell and (**B**) grain size dependence, respectively, for Co-, Mn-, and Ni-doped magnetite structure.

The XRD patterns allow for an estimation of the oxidation degree of magnetite/maghemite to hematite phase. The obtained diffractograms are depicted in Figure 2 and Figure 3. Table 2 and Figure 4 present the grain size and strain of nanoparticles crystals as calculated by the Williamson–Hall equation [29]. Moreover, lattice cell parameters of magnetite/maghemite, and hematite (if present), as well as volume/mass ratios of magnetite/maghemite to hematite phases were evaluated.

The diffraction patterns presented in Figure 2 exhibit, in all cases, a typical magnetite/maghemite inverse spinel structure before thermal treatment. These peaks can be assigned by Miller indexes as (220), (311), (400), (422), (511), and (440) [30,31]. Signals which are typical for a hematite structure can be seen only in the case of Co- and Mn-doped nanoparticles after heating at 500 °C. Interpreted spectra reveal that the lattice constants of magnetite and hematite are close to theoretical bulk values (8.39 Å [32] for magnetite, 8.35 Å [33] for maghemite, and 5.03 Å [34] for hematite). In addition, due to the size- and composition-related broadening of reflexes, magnetite and maghemite are indistinguishable by XRD analysis only. However, in Figure 2D–F, an evident shift toward higher 2θ value, which is related with cell size shrinkage, is observed. That indicates a developed intermediate state in the oxidation pathway from magnetite-like via maghemite-like to hematite-like structures. In Figure 3, only the peaks of a magnetite-like structure can be recognized in the diffractograms. The concentration of the doping element was slightly higher compared to Figure 2 and thus blocked the oxidation to hematite. 

The diffractograms depicted in Figure 2 and Figure 3 and the fitted crystal parameters summarized in Table 2 indicate that oxidation degree can be studied by XRD to some extent. In the case of Co-doped nanoparticles, a small amount of doping (up to 15%) causes the occurrence of a hematite phase, contributing to approximately 25% of the sample volume. The oxidation is much stronger in 0.1 doping case, which is contrary to the observations mentioned above. This particular case has to be studied in more detail due to the extraordinary behavior that was seen. At a higher magnetite doping percentage (20% by Co), only a small fraction (<2%) of the nanoparticles can be oxidized, which is invisible to X-ray diffraction. A similar effect can be observed in the case of Mn-doped nanoparticles. Here, the smallest amount of Mn (5%) causes oxidation of up to 36%. However, with an increase in Mn content, the tendency of thermal oxidation significantly decreases. The presence of Ni in magnetite structure triggers oxidation to the hematite and limits it to a maximum 8% level in all cases (Table 2).

**Figure 5:**
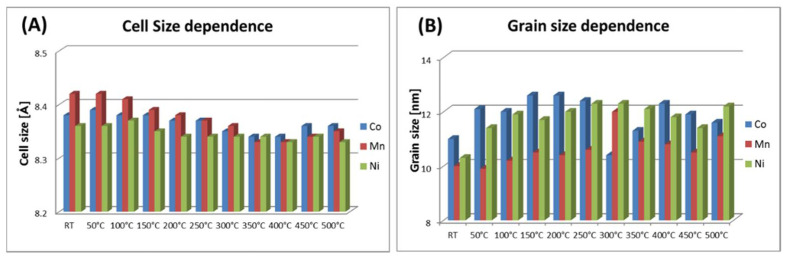
Bar plots of nanoparticles’ (**A**) cell and (**B**) grain size dependence for whole temperature range (RT–500 °C) for Co-, Mn-, and Ni- x = 0.20 doped magnetite structures.

The average particle sizes estimated by XRD show that heat treatment had no significant influence on this parameter within the uncertainty limits. Thermally induced particle size growth was not observed, which is often suggested in the literature [35]. Specifically, strong stability of particles’ size studied in series is seen when it is calculated and plotted every 50 °C (Table 3, Figure 5). From such presentation, temperature tendency can be summarized as follows: average cell size decreases in accordance with slow oxidation to maghemite (Figure 5A); average grain size shows a slow increasing trend with small disturbance around 300 °C for Co and Mn, and in the case of Ni, gradual increment in grain size is observed.

The replacement of iron in the magnetite/maghemite crystal structure by another *3d* element (e.g., Co, Mn, Ni) in restricted amounts causes a much lower susceptibility to oxidation in comparison to undoped magnetite/maghemite [28]. The degree of this effect, however, is not the same for every case. Ni-doped ferrite nanoparticles are the most resistant to oxidation while those doped with Co are the least resistant. In addition, the evaluation of average cell parameters for thermally treated series shows clearly that magnetite-like values (for RT) change, on average, to lower (for 500 °C) ones, typical for maghemite. This trend is in agreement with the bulk transformation scheme, where a direct oxidation from magnetite to hematite is very unlikely. Maghemite as a transitional phase is always present. Moreover, our results show that the presence of extraordinary Fe-oxides such as β- or ε-Fe_2_O_3_ phases [36] was not observed. 

### 3.3. Infrared Spectroscopy (IR)

**Figure 6:**
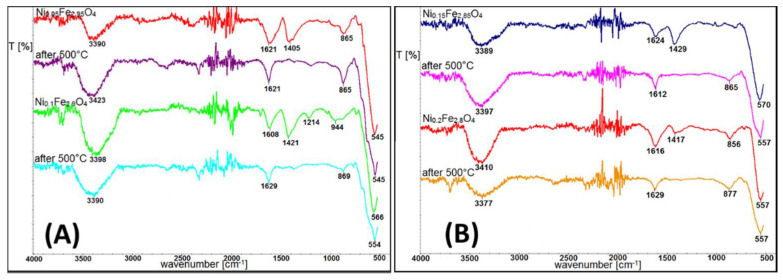
Infrared spectra of Ni-doped nanoparticles (**A**) before and (**B**) after heat treatment.

In order to reveal surface modifications after exposure to high temperature, FTIR experiments were performed. Spectra of Ni-doped ferrites before and after temperature treatment are presented in Figure 6. For each doping series, the spectra behave in the same manner; therefore, the Ni case was selected as an example.

From the selected FTIR spectra, it can be seen that the most pronounced surface modification received by IR is connected with water evaporation and reduction of the O–H bond signal. Bands around 1400 cm^−1^, which are typical for O–H bonds, disappear after heat treatment. Fe–O bonds in magnetite and maghemite lie very close to each other [37,38]. However, a small shift towards higher cm^−1^ after heating at 500 °C can be noticed. Therefore such modification of the peak shape below 590 cm^−1^ indicates presence of magnetite and that is related to maghemite oxidation. Band positions typical for C–H in TBAOH used as a surfactant remain unchanged (signals at around 1620 cm^−1^ and 865 cm^−1^) but its relative intensity is lower in comparison to pristine samples. Vibrations around 3300 cm^−1^ typical for O–H bonds in water and TBAOH are still visible, even after high temperature treatment.

### 3.4. Differential Scanning Calorimetry (DSC)

**Figure 7:**
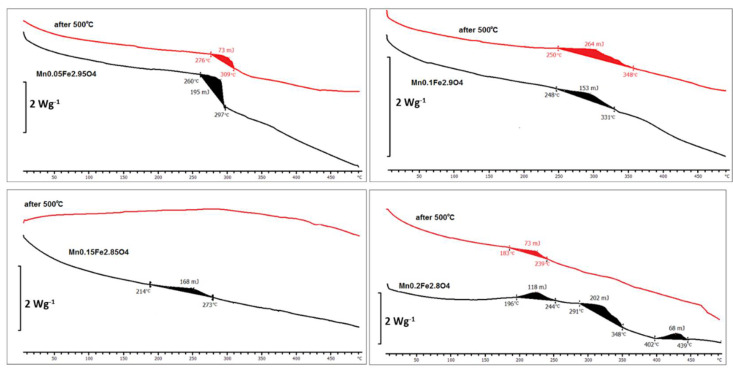
Differential scanning calorimetry (DSC) measurements of Mn-doped nanoparticles before and after thermal treatment.

A thermal analysis of ferrite nanoparticles was conducted in a quick heating and cooling cycle in the temperature range of 2–450 °C with scan rate of 10 °C/min. As reference, an empty pan kept in the same condition was used. For the measurement, a quantity of roughly 2 mg of the total weight was separated. The obtained curves are depicted in Figure 7.

Figure 7 presents a selected (Mn) set of DSC thermal analysis curves. Particles with small doping M_x_ ≤ 0.1 before and after heat treatment changed in very similar ways (exothermic process < 264 J between 248 and 348 °C). Only after threshold of x = 0.15 does their sensitivity to heat start to be dependent on heating history (as prepared and after exposure to 500 °C). In all cases, a transition of the same nature takes place in the temperature range 180–450 °C (not very intensive exothermic process).

### 3.5. Vibrating Sample Magnetometry (VSM)

A subset of the investigated samples (Mn-doped ferrites) was characterized by vibrating sample magnetometry (VSM), and the most important parameters were calculated from magnetization curves. Values of blocking temperature (T_bm_), remanence (M_rs_), saturation magnetization (M_s_), and coercive field (H_c_) are presented in Table 4.

**Figure 8:**
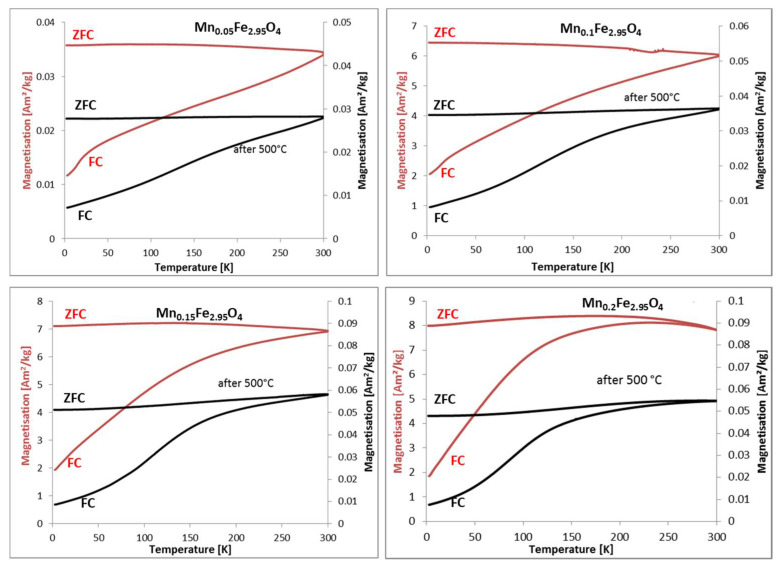
ZFC and FC curves of Mn-doped ferrite nanoparticles of different doping degrees before (black), and after treatment at 500 °C (red).

**Figure 9:**
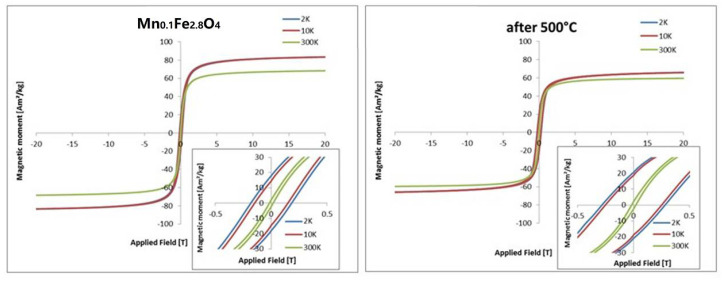
Hysteresis loops of Mn_0.15_Fe_2.85_O_4_ nanoparticles at 2, 10, and 300 K before and after heating to 500 °C.

It can be seen that the selected series of magnetization curves (Figure 8) indicate that heat treatment influences the magnetization parameters. In general, remanence is almost unchanged, but saturation magnetization decreases while coercive field increases after exposure to 500 °C (Table 4). It is also clear that the blocking temperature lies below 300 K in the case of Mn0.2 and, above, in all other cases adopting the Hansen and Mørup model [39]. The presented hysteresis curves (Figure 9) is able to measure the coercive field up to 300 K. However, the opening of the hysteresis loop is best observable at low temperatures. In general, magnetic properties’ characterization confirms the appearance of new-formed phases after heat treatment. These are responsible for the significant decrease in the saturation magnetization that can also be observed at room temperature in contrast to the coercive field Hc. Such changes in VSM indicate that transformation into a maghemite-like phase is the intermediate state before α-Fe_2_O_3_ can be achieved as a final form.

### 3.6. Mössbauer Spectroscopy

**Figure 10:**
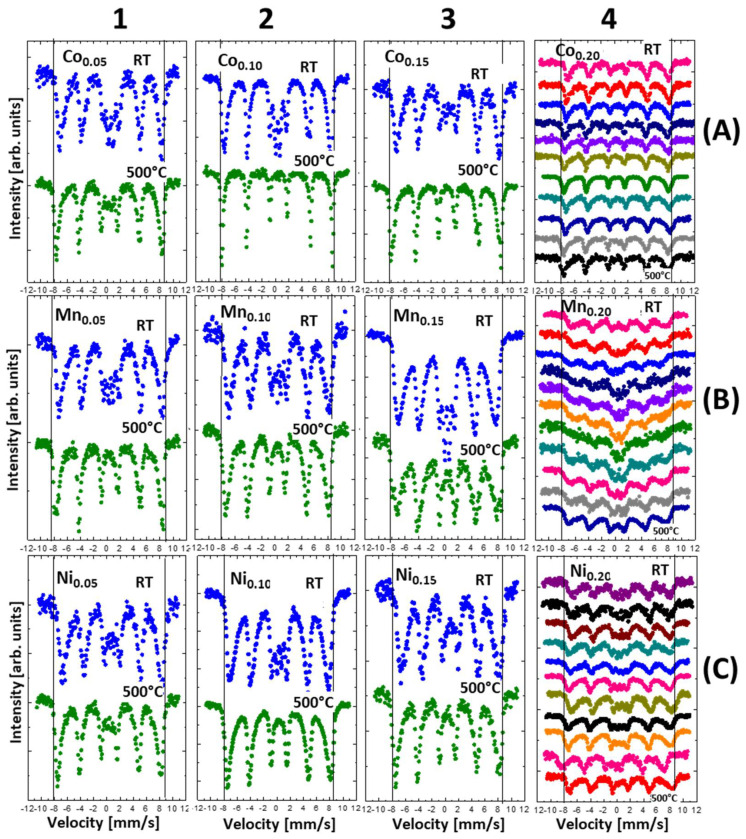
Mössbauer spectra of ferrite nanoparticles before (Ref.) and after (500 °C) thermal treatment procedure ((**A**) Co, (**B**) Mn, and (**C**) Ni or (**1**) 0.05, (**2**) 0.1, (**3**) 0.15, and (**4**) 0.2). The vertical line represents the position of the first and last lines of the bulk hematite Mössbauer spectrum on the velocity scale.

The oxidation effect of thermally treated nanoparticles was also investigated by Mössbauer spectroscopy. The measurements were carried out at room temperature using a ^57^Co source. All discussed nanoparticles before and after heat treatment were measured in 1 cm^2^ tablet form mixed with boron nitride (BN) as a support. In Figure 10, the summarized spectra are presented in proper series. The results are sorted in series, with respect to the amount of doping (windows 1, 2, 3, and 4) and type of the doping element (Figure 10A—Co; B—Mn; C—Ni ions). The last column presents the series for all measured spectra after thermal treatment in the temperature range of 50–500 °C in increments of 50 °C.

In Figure 10, rows A, B, and C depict Mössbauer spectra series of Co, Mn, and Ni, respectively. In general, the spectra measured without thermal treatment can be described by three sub-spectra: two sextets which correspond to magnetite tetrahedral Fe[A] and octahedral Fe[B] position with a hyperfine magnetic field equal to (49 ± 1) T and (45 ± 1) T [40], respectively, and a superparamagnetic doublet present in the central part of spectra. If heating-induced oxidation starts, magnetite transforms into maghemite, as characterized by two sextets with almost the same hyperfine parameters and with hyperfine magnetic field (HMF) of about (50 ± 1) T. Further oxidation causes an additional sextet with a hyperfine magnetic field of (52 ± 1) T, which originates from the iron atoms in the hematite phase. The process of oxidation of nanoparticles is observed in the spectra lines by increasing both an average hyperfine magnetic field and intensities of outermost sextet. Because the spectral line widths of the sextets are broad, the magnetite, maghemite, and hematite components are not well resolved [41]. For this reason, quantitative observation of the increasing intensity of the maghemite or hematite sextet during oxidation is difficult. The oxidation can be much better observed by another Mössbauer parameter: the increase in the average hyperfine magnetic field. The black lines in the frames in Figure 10 represent reference points, while the position of the first and last lines of the sextet are attributed to the iron in the hematite phase. In the case of Co substitution (x = 0.05, 0.1, 0.15, 0.2), the samples without thermal treatment can be described by their magnetite and superparamagnetic components. After thermal treatment, the hyperfine magnetic field increases with the rising heating temperature. Moreover, the intensity of the superparamagnetic doublet decreases with increasing temperature. For example, where x = 0.1, 0.15, 0.2, this component completely disappears. In the case of Mn substitution, the Mössbauer spectra of reference samples can be described in the same manner as in the case of Co. After thermal treatment, the hyperfine magnetic field increases, but not as clearly as in the Co series. One can conclude that Mn atoms substituted in magnetite cause resistance to oxidation. For Ni substitution, there is no clear elevation of the HMF with increasing heat treatment temperature. All measured Mössbauer spectra are broad and do not change after oxidation, except for the disappearance of the superparamagnetic component at x = 0.15. It seems that for this substitution, the magnetite is most resistant to oxidation.

As one can see in Figure 10 column 4, the shapes of Mössbauer spectra behave in different ways as a function of annealing temperature. In the case of Co substitution, the spectra are relatively sharp. For Ni substitution, the spectra are broader compared to Co dopant and doublets are seen in the central part of measured spectra. Different behavior is observed in the case of Mn substitution. For Mn, spectra are very broad for all temperature treatment, especially for T = 300 °C, and it looks like superparamagnetic behavior. Most probably, such behavior can be attributed to the magnetic properties of dopants. Cobalt and Ni ferrites show ferromagnetic behaviors, while the Mn ferrite contains considerable antiferromagnetic contributions. Presumably, substitution of Mn in magnetite causes a decrease in blocking temperature T_B_.

## 4. Conclusions

The data presented in this paper indicate that doping of magnetite by *3d* elements influences its reactivity/resistance to the oxidative environment very strongly. However, each case (element) has to be treated separately since their endurance to the oxidation process is not the same, as shown by XRD and MS. The particular application has to be taken into account when particles are designed. Nevertheless, the study shows that particles on demand can be obtained with success and their durability against external stimuli depends very strongly on their initial composition.

The following conclusions can be drawn from the presented measurements and their analyses:

- For the application of nanoparticles in an environment where rising temperature is expected, doping of the inorganic core by Co, Mn, and Ni is useful. Doping prevents the spontaneous oxidation induced by high temperatures. The results reveal that Ni doping can even suppress the oxidation process.
- Particles exposed regularly or for a long period of time to high temperatures should be tested for their magnetic properties before and after heat treatment. It was observed that slow transformation from one oxide to another causes significant magnetic property changes, particularly saturation magnetization.
- Each of the studied oxides has its own magnetic characteristic; therefore, they cannot be expected to behave the same way when the application is considered.

## Figures and Tables

**Table 1:** Type of obtained nanoparticles and their abbreviations used throughout the paper. Selected samples of Fe and M (Co, Mn, Ni) concentrations were obtained from energy dispersive X-ray (EDX) analysis.

Type of Nanoparticle	Abbreviation	%Fe		%M	
		Weight ± 2%	Theory	Weight ± 2%	Theory
**Co_0.05_Fe_2.95_O_4_**	Co_0.05_	94	95	6	5
**Co_0.1_Fe_2.9_O_4_**	Co_0.1_	91	92	9	8
**Co_0.15_Fe_2.85_O_4_**	Co_0.15_	87	88	13	12
**Co_0.2_Fe_2.8_O_4_**	Co_0.2_	81	84	19	16
**Mn_0.05_Fe_2.95_O_4_**	Mn_0.05_	93	96	7	4
**Mn_0.1_Fe_2.9_O_4_**	Mn_0.1_	89	92	11	8
**Mn_0.15_Fe_2.85_O_4_**	Mn_0.15_	83	88	17	12
**Mn_0.2_Fe_2.8_O_4_**	Mn_0.2_	77	85	22	15
**Ni_0.05_Fe_2.95_O_4_**	Ni_0.05_	94	96	6	4
**Ni_0.1_Fe_2.9_O_4_**	Ni_0.1_	90	92	10	8
**Ni_0.15_Fe_2.85_O_4_**	Ni_0.15_	85	88	15	12
**Ni_0.2_Fe_2.8_O_4_**	Ni_0.2_	80	85	20	15

**Table 2:** The following crystallographic parameters of nanoparticles, with different degrees of metal doping, were determined by XRD: grain size, strain, lattice cell parameters of magnetite and hematite (if present), as well as volume percentage contribution of both phases. The parameters for stoichiometric magnetite are given for comparison (a.p. stands for as prepared).

Name	Size (nm) ± 1	Strain [10^3^] ± 0.5	Fe_3_O_4_ Cell Size (Å) ± 0.02	Fe_2_O_3_ Cell Size (Å) ± 0.02	Fe_3_O_4_ (%) ± 1	α-Fe_2_O_3_ (%) ± 1
**Fe_3_O_4_**	15	3.1	8.37	-	100	0
**Co_0.05_ a.p.**	13	3.7	8.38	-	100	0
**Co_0.05_ 500 °C**	15	4.1	8.34	5.03	77	23
**Co_0.10_ a.p.**	13	4.1	8.37	5.03	78	22
**Co_0.10_ 500 °C**	25	5.0	8.37	5.04	25	75
**Co_0.15_ a.p.**	13	3.1	8.39	-	100	0
**Co_0.15_ 500 °C**	15	3.7	8.37	5.04	76	24
**Mn_0.05_ a.p.**	15	4.4	8.39	-	80	20
**Mn_0.05_ 500 °C**	18	4.3	8.34	5.03	64	36
**Mn_0.10_ a.p.**	13	3.1	8.42	-	100	0
**Mn_0.10_ 500 °C**	15	4.0	8.34	5.04	85	15
**Mn_0.15_ a.p.**	12	4.5	8.42	-	100	0
**Mn_0.15_ 500 °C**	12	3.8	8.35	5.03	86	14
**Ni_0.05_ a.p.**	13	3.0	8.37	-	100	0
**Ni_0.05_ 500 °C**	14	3.4	8.34	5.03	94	6
**Ni_0.10_ a.p.**	11	1.9	8.37	-	100	0
**Ni_0.10_ 500 °C**	13	3.2	8.34	5.04	92	8
**Ni_0.15_ a.p.**	12	3.1	8.37	-	100	0
**Ni_0.15_ 500 °C**	13	3.2	8.34	5.03	96	4

**Table 3:** The following crystallographic parameters of nanoparticles with a constant degree of metal doping were determined by XRD as a function of heat treatment temperature: grain size, strain, lattice constant. Here, only Fe_3_O_4_ phase was detected, and no traces of α-Fe_2_O_3_ phase (a.p.—as prepared).

Name	Size (nm) ± 1	Strain [10^3^] ± 0.5	Fe_3_O_4_ Cell Size (Å) ± 0.02
**Co_0.20_ a.p.**	11	2.1	8.38
**Co_0.20_ 50 °C**	12	2.1	8.39
**Co_0.20_ 100 °C**	12	1.2	8.38
**Co_0.20_ 150 °C**	13	3.2	8.38
**Co_0.20_ 200 °C**	13	3.2	8.37
**Co_0.20_ 250 °C**	12	2.8	8.37
**Co_0.20_ 300 °C**	10	4.7	8.35
**Co_0.20_ 350 °C**	11	1.7	8.34
**Co_0.20_ 400 °C**	12	3.1	8.34
**Co_0.20_ 450 °C**	12	2.5	8.36
**Co_0.20_ 500 °C**	12	1.7	8.36
**Mn_0.20_ a.p.**	10	5.1	8.42
**Mn_0.20_ 50 °C**	10	4.7	8.42
**Mn_0.20_ 100 °C**	10	4.5	8.41
**Mn_0.20_ 150 °C**	11	4.6	8.39
**Mn_0.20_ 200 °C**	10	4.7	8.38
**Mn_0.20_ 250 °C**	11	4.7	8.37
**Mn_0.20_ 300 °C**	12	2.1	8.36
**Mn_0.20_ 350 °C**	11	3.9	8.33
**Mn_0.20_ 400 °C**	11	3.4	8.33
**Mn_0.20_ 450 °C**	11	3.5	8.34
**Mn_0.20_ 500 °C**	11	3.9	8.35
**Ni_0.20_ a.p.**	10	2.8	8.36
**Ni_0.20_ 50 °C**	11	2.4	8.36
**Ni_0.20_ 100 °C**	12	2.8	8.37
**Ni_0.20_ 150 °C**	12	2.4	8.35
**Ni_0.20_ 200 °C**	12	2.4	8.34
**Ni_0.20_ 250 °C**	12	3.1	8.34
**Ni_0.20_ 300 °C**	12	3.1	8.34
**Ni_0.20_ 350 °C**	12	2.8	8.34
**Ni_0.20_ 400 °C**	12	2.5	8.33
**Ni_0.20_ 450 °C**	11	1.7	8.34
**Ni_0.20_ 500 °C**	12	2.6	8.33

**Table 4:** Magnetization values calculated from Field Cooled/Zero Field Cooled (FC/ZFC) and hysteresis curves. T_bm_—superparamagnetic blocking temperature (H–M—Hansen and Mørup model; M—Micha et al. model); M_rs_—remanence; M_s_—saturation magnetization; H_c_—coercive field; χ_hf_—high field susceptibility. Column MS describes blocking temperatures determined from Mössbauer spectroscopy.

Sample	MS	T_bm_ (K) ± 1		M_rs_ (A·m^2^/kg) ± 0.5			M_s_ (A·m^2^/kg) ± 0.5			H_c_ (mT) ± 0.5		
		H-M	M	2 K	10 K	300 K	2 K	10 K	300 K	2 K	10 K	300 K
**Mn_0.20_**	>RT	169	5.6	28.4	20.5	0.2	88.6	88.7	68.1	29.4	19.7	0.1
**Mn_0.20_ 500 °C**	>RT	243	84.9	27.9	25.4	0.1	65.6	65.6	55.3	44.7	38.4	0.1
**Mn_0.15_**	>RT	>300	299	22.7	18.1	0.6	82.4	82.5	67.0	23.3	18.0	0.4
**Mn_0.15_ 500 °C**	>RT	>300	113	23.0	21.5	0.3	57.9	57.7	50.7	38.3	34.4	0.2
**Mn_0.10_**	>RT	>300	299	20.9	17.9	2.1	85.4	85.4	69.7	21.5	18.2	1.6
**Mn_0.10_ 500 °C**	>RT	>300	116	23.2	21.4	1.7	68.3	68.3	60.1	31.5	28.5	1.4
**Mn_0.05_**	>RT	>300	299	17.8	15.3	3.6	68.1	68.0	59.1	24.4	20.8	3.5
**Mn_0.05_ 500 °C**	>RT	>300	299	18.4	17.6	3.5	53.4	53.4	47.2	30.8	29.2	3.6
**Co_0.05_**	>RT	>300	299	50.8	49.5	11.3	70.3	70.2	59.7	512	480	23.0
**Ni_0.05_**	>RT	>300	299	22.7	19.9	2.8	76.5	76.9	70.1	24.2	21.1	2.0

## Data Availability

The raw data required to reproduce these findings cannot be shared at this time as the data also forms part of an ongoing study.

## References

[1] Wang C., Meyer J., Teichert N., Auge A., Rausch E., Balke B., Hütten A., Fecher G.H., Felser C. (2014). Heusler nanoparticles for spintronics and ferromagnetic shape memory alloys. J. Vac. Sci. Technol. B.

[2] Kefeni K.K., Msagati T.A.M., Mamba B.B. (2017). Ferrite nanoparticles: Synthesis, characterisation and applications in electronic device. Mater. Sci. Eng. B.

[3] Wang L., Ma W., Xu L., Chen W., Zhu Y., Xu C., Kotov N.A. (2010). Nanoparticle-based environmental sensors. Mater. Sci. Eng. R Rep..

[4] Cowburn R.P., Welland M.E. (2000). Room Temperature Magnetic Quantum Cellular Automata. Science.

[5] Patitsa M., Karathanou K., Kanaki Z., Tzioga L., Pippa N., Demetzos C., Verganelakis D.A., Cournia Z., Klinakis A. (2017). Magnetic nanoparticles coated with polyarabic acid demonstrate enhanced drug delivery and imaging properties for cancer theranostic applications. Sci. Rep..

[6] Amiri M., Akbari A., Ahmadi M., Pardakhti A., Salavati-Niasari M. (2018). Synthesis and in vitro evaluation of a novel magnetic drug delivery system; proecological method for the preparation of CoFe_2_O_4_ nanostructures. J. Mol. Liq..

[7] Naahidi S., Jafari M., Edalat F., Raymond K., Khademhosseini A., Chen P. (2013). Biocompatibility of engineered nanoparticles for drug delivery. J. Control. Release.

[8] Haun J.B., Yoon T.J., Lee H., Weissleder R. (2010). Magnetic nanoparticle biosensors. Wiley Interdiscip. Rev. Nanomed. Nanobiotechnol..

[9] Hedayatnasab Z., Abnisa F., Daud W.M.A.W. (2017). Review on magnetic nanoparticles for magnetic nanofluid hyperthermia application. Mater. Des..

[10] Mody V.V., Cox A., Shah S., Singh A., Bevins W., Parihar H. (2013). Magnetic nanoparticle drug delivery systems for targeting tumor. Appl. Nanosci..

[11] Périgo E.A., Hemery G., Sandre O., Ortega D., Garaio E., Plazaola F., Teran F.J. (2015). Fundamentals and advances in magnetic hyperthermia. Appl. Phys. Rev..

[12] Sahoo Y., Goodarzi A., Swihart M.T., Ohulchanskyy T.Y., Kaur N., Furlani E.P., Prasad P.N. (2005). Aqueous ferrofluid of magnetite nanoparticles: Fluorescence labeling and magnetophoretic control. J. Phys. Chem. B.

[13] Zakharova I.N., Shipilin M.A., Alekseev V.P., Shipilin A.M. (2012). Mössbauer study of maghemite nanoparticles. Tech. Phys. Lett..

[14] Chourpa I., Douziech-Eyrolles L., Ngaboni-Okassa L., Fouquenet J.-F., Cohen-Jonathan S., Soucé M., Marchais H., Dubois P. (2005). Molecular composition of iron oxide nanoparticles, precursors for magnetic drug targeting, as characterized by confocal Raman microspectroscopy. Analyst.

[15] Issa B., Obaidat I.M., Albiss B.A., Haik Y. (2013). Magnetic nanoparticles: Surface effects and properties related to biomedicine applications. Int. J. Mol. Sci..

[16] Gossuin Y., Gillis P., Hocq A., Vuong Q.L., Roch A. (2009). Magnetic resonance relaxation properties of superparamagnetic particles. Nanomed. Nanobiotechnol..

[17] Amiri M., Pardakhti A., Ahmadi-Zeidabadi M., Akbari A., Salavati-Niasari M. (2018). Magnetic nickel ferrite nanoparticles: Green synthesis by Urtica and therapeutic effect of frequency magnetic field on creating cytotoxic response in neural cell lines. Colloids Surf. B Biointerfaces.

[18] Ali A., Zafar H., Zia M., Haq I.U., Phull A.R., Ali J.S., Hussain A. (2016). Synthesis, characterization, applications, and challenges of iron oxide nanoparticles. Nanotechnol. Sci. Appl..

[19] Mamiya H., Ohnuma M., Nakatani I., Furubayashim T. (2005). Extraction of blocking temperature distribution from zero-field-cooled and field-cooled magnetization curves. IEEE Trans. Magn..

[20] Reddy L.H., Arias J.L., Nicolas J., Couvreur P. (2012). Magnetic nanoparticles: Design and characterization, toxicity and biocompatibility, pharmaceutical and biomedical applications. Chem. Rev..

[21] Petran A., Radu T., Borodi G., Nan A., Suciu M., Turcu R. (2018). Effects of rare earth doping on multi-core iron oxide nanoparticles properties. Appl. Surf. Sci..

[22] Kowalik P., Mikulski J., Borodziuk A., Duda M., Kamińska I., Zajdel K., Rybusinski J., Szczytko J., Wojciechowski T., Sobczak K. (2020). Yttrium-Doped Iron Oxide Nanoparticles for Magnetic Hyperthermia Applications. J. Phys. Chem. C.

[23] Sivagurunathan P., Sathiyamurthy K. (2016). Effect of Temperatures on Structural, Morphological and Magnetic Properties of Zinc Ferrite Nanoparticles. Can. Chem. Trans. Year.

[24] Tuček J., Zbořil R., Namai A., Ohkoshi S. (2010). ε-Fe_2_O_3_: An Advanced Nanomaterial Exhibiting Giant Coercive Field, Millimeter-Wave Ferromagnetic Resonance, and Magnetoelectric Coupling. Chem. Mater..

[25] Kalska-Szostko B., Kropiewnicka K. (2012). The influence of the transition metal substitution on chemically prepared ferrite nanoparticles—Mossbauer studies. Curr. Appl. Phys..

[26] Colombo M., Carregal-Romero S., Casula M.F., Gutiérrez L., Morales M.P., Böhm I.B., Heverhagen J.T., Prosperi D., Parak W.J. (2012). Biological applications of magnetic nanoparticles. Chem. Soc. Rev..

[27] Sun Y.K., Ma M., Zhang Y., Gu N. (2004). Synthesis of nanometer-size maghemite particles from magnetite. Colloids Surf. A Physicochem. Eng. Asp..

[28] Kalska-Szostko B., Wykowska U., Satula D., Nordblad P. (2015). Thermal treatment of magnetite nanoparticles. Beilstein J. Nanotechnol..

[29] Mote V., Purushotham Y., Dole B. (2012). Williamson-Hall analysis in estimation of lattice strain in nanometer-sized ZnO particles. J. Theor. Appl. Phys..

[30] Mandal M., Kundu S., Ghosh S.K., Panigrahi S., Sau T.K., Yusuf S.M., Pal T. (2005). Magnetite nanoparticles with tunable gold or silver shell. J. Colloid Interface Sci..

[31] Kalska-Szostko B., Wykowska U., Satuła D. (2015). Magnetic nanoparticles of core-shell structure. Colloids Surf. A Physicochem. Eng. Asp..

[32] Panda R.N., Gajbhiye N.S., Balaji G. (2001). Magnetic properties of interacting single domain Fe_3_O_4_ particles. J. Alloys Compd..

[33] Tronc E., Ezzir A., Cherkaoui R., Chaneac C., Nogues M., Kachkachi H., Fiorani D., Testa A.M., Greneche J.M., Jolivet J.P. (2000). Surface-related properties of gamma-Fe_2_O_3_ nanoparticles. J. Magn. Magn. Mater..

[34] Chen T., Xu H., Xie Q., Chen J., Ji J., Lu H. (2005). Characteristics and genesis of maghemite in Chinese loess and paleosols: Mechanism for magnetic susceptibility enhancement in paleosols. Earth Planet. Sci. Lett..

[35] Singh L.H., Pati S.S., Sales M.J.A., Guimarães E.M., Oliveira A.C., Garg V.K. (2015). Facile Method to Tune the Particle Size and Thermal Stability of Magnetite Nanoparticles. J. Braz. Chem. Soc..

[36] Vujtek M., Zboril R., Kubinek R., Mashlan M. (2003). Ultrafine Particles of Iron(III) Oxides by View of AFM—Novel Route for Study of Polymorphism in Nano-world. Sci. Technol. Educ. Microsc..

[37] Balasubramaniam R., Ramesh Kumar A.V. (2000). Characterization of Delhi iron pillar rust by X-ray diffraction, Fourier transform infrared spectroscopy and Mossbauer spectroscopy. Corros. Sci..

[38] Kalska-Szostko B., Wykowska U., Piekut K., Satuła D. (2014). Stability of Fe_3_O_4_ nanoparticles in various model solutions. Colloids Surf. A Physicochem. Eng. Asp..

[39] Hansen M.F., Mørup S. (1999). Estimation of blocking temperatures from ZFC/FC curves. J. Magn. Magn. Mater..

[40] Kalska-Szostko B., Cydzik M., Satuła D., Giersig M. (2011). Mössbauer Studies of Core-Shell Nanoparticles. Acta Phys. Pol. A.

[41] Kalska-Szostko B., Satuła D., Olszewski W. (2015). Mössbauer spectroscopy studies of the magnetic properties of ferrite nanoparticles. Curr. Appl. Phys..

